# Evolution of motif variants and positional bias of the cyclic-AMP response element

**DOI:** 10.1186/1471-2148-7-S1-S15

**Published:** 2007-02-08

**Authors:** Brandon Smith, Hung Fang, Youlian Pan, P Roy Walker, A Fazel Famili, Marianna Sikorska

**Affiliations:** 1 Neurogenomics Group, Institute for Biological Sciences, National Research Council of Canada, Ottawa, Ontario, Canada; 2 Glycosyltransferases and Neuroglycomics Group, Institute for Biological Sciences, National Research Council of Canada Ottawa Ontario, Canada; 3 Neurogenesis and Brain Repair Group, Institute for Biological Sciences, National Research Council of Canada, Ottawa, Ontario, Canada; 4 Integrated Reasoning Group, Institute for Information Technology, National Research Council of Canada, Ottawa, Ontario, Canada

## Abstract

**Background:**

Transcription factors regulate gene expression by interacting with their specific DNA binding sites. Some transcription factors, particularly those involved in transcription initiation, always bind close to transcription start sites (TSS). Others have no such preference and are functional on sites even tens of thousands of base pairs (bp) away from the TSS.

The Cyclic-AMP response element (CRE) binding protein (CREB) binds preferentially to a palindromic sequence (TGACGTCA), known as the canonical CRE, and also to other CRE variants. CREB can activate transcription at CREs thousands of bp away from the TSS, but in mammals CREs are found far more frequently within 1 to 150 bp upstream of the TSS than in any other region. This property is termed positional bias.

The strength of CREB binding to DNA is dependent on the sequence of the CRE motif. The central CpG dinucleotide in the canonical CRE (TGA**CG**TCA) is critical for strong binding of CREB dimers. Methylation of the cytosine in the CpG can inhibit binding of CREB. Deamination of the methylated cytosines causes a C to T transition, resulting in a functional, but lower affinity CRE variant, TGA**T**GTCA.

**Results:**

We performed genome-wide surveys of CREs in a number of species (from worm to human) and showed that only vertebrates exhibited a CRE positional bias. We performed pair-wise comparisons of human CREs with orthologous sequences in mouse, rat and dog genomes and found that canonical and TGA**T**GTCA variant CREs are highly conserved in mammals. However, when orthologous sequences differ, canonical CREs in human are most frequently TGA**T**GTCA in the other species and vice-versa. We have identified 207 human CREs showing such differences.

**Conclusion:**

Our data suggest that the positional bias of CREs likely evolved after the separation of urochordata and vertebrata. Although many canonical CREs are conserved among mammals, there are a number of orthologous genes that have canonical CREs in one species but the TGA**T**GTCA variant in another. These differences are likely due to deamination of the methylated cytosines in the CpG and may contribute to differential transcriptional regulation among orthologous genes.

## Background

Identification of transcription factor binding sites is crucial to the understanding of gene regulation at the transcriptional level and for deciphering gene regulatory networks. In recent years, the power of bioinformatics and the emergence of complete genome sequences from a variety of species have made it possible to perform global *in silico *surveys for putative binding sites of individual transcription factors.

The Cyclic-AMP response element binding protein (CREB) belongs to the bZip family of transcription factors that contain basic leucine zipper motifs. CREB activates target genes by binding to the cAMP-response element (CRE) most frequently located in the promoter region [1]. Examples of bZip proteins binding to CRE and CRE-like motifs have been described in eukaryotes from yeast to human [1,2]. In mammals, CREB plays an important role in many biological processes [1] and can be activated through several signaling pathways (see reviews: [1,3]). For instance, CREB has been implicated in multiple functions essential to the brain, including responses to emotional stimuli [4], learning and long-term memory formation [5-9]. Dysfunction in CREB-regulated transcription also contributes to neurodegeneration [10-12]. The canonical CRE is a palindromic octamer, TGACGTCA. However, degenerate CREs and half-CREs (TGACG) are also found to be functional in many of the CREB targets identified so far [1], suggesting a tolerance of CREB for CREs in terms of sequence recognition and binding. More than 100 mammalian CREB target genes have been identified to date (reviewed in [1,13]), and there are thousands of putative CREB binding sites that are likely to be functional [14,15].

It has been observed that, in mammalian genomes, the canonical CRE shows a positional bias [16] towards the proximal promoter region [15,17], although it remains unclear how this feature has evolved. Mammalian genomes, as is typical of vertebrate genomes, are globally methylated [18] and contain many CpG islands [19,20]. CpG islands are 200 bp or longer DNA regions with GC-content greater than 50% and a higher than expected number of CpG dinucleotides [21]. Approximately 60 percent of human genes (including most, if not all, house-keeping genes) have CpG islands in their promoters [22]. Most CpG dinucleotides in mammalian genomes contain 5-methylcytosine [22]. However, although CpG dinucleotides in CpG islands can be methylated, they are typically maintained in an unmethylated state [22]. CpGs are usually methylated on both complementary strands, but are sometimes maintained in a hemi-methylated state [23]. The central dinucleotide of the canonical CRE motif, CpG, is known to be methylated in some cases [14]. Such methylation inhibits CREB binding [14] and presumably also increases the rate of C to T transition due to spontaneous deamination of methyl-C. These transitions are often not corrected by DNA repair mechanisms and can be retained as single nucleotide polymorphisms that may cause altered gene expression and changes in phenotype. These transitions may persist through evolution, potentially resulting in differences in CRE function in orthologous genes. CREs with a central TpG (or CpA) have lower affinity for CREB binding compared to the canonical CRE [24,25], but have been shown to be functional in some genes [25-29]. Deamination of both complements of a fully methylated CpG dinucleotide results in a change from CpG to TpA. The TGA**TA**TCA motif is not known to function as a CRE.

In this study, we performed genome-wide searches for the canonical CRE and a variant CRE motif (TGA**T**GTCA, called the TG-variant in this work) in promoter regions of human, mouse, rat, dog, chicken, fish, frog, fruit fly, sea squirt and worm, to determine if the positional bias of CREs is present in other genomes and to establish the point in evolutionary history that the positional bias of CREs started to appear. We also performed comparative genomics analyses to investigate the conservation of orthologous CRE motifs in human, mouse, rat and dog. We focused on differences in the central dinucleotide of the CRE motifs, which is critical for strong CREB binding. Since, in mammals, methylation of sites other than the CpG dinucleotides is rare, TG-variant CREs are not expected to be affected by methylation mechanisms. Using pairwise comparisons of human CREs with orthologous sequences in mouse, rat and dog, we identified known and putative CREs that show CpG to TpG transitions in the core of the CRE motif between orthologous genes. Such differences between CREs in the promoter regions of orthologous genes may cause significant differences in temporal or tissue specific expression in these orthologs.

## Results and Discussion

### Positional bias of CRE-like motifs

**Figure 1:**
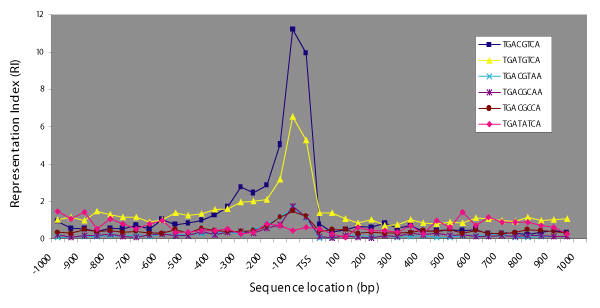
**Variation of representation index (RI) of CRE motifs over human TSS-SR regions**. The representation index of each 50 bp window along the human TSS-SRs are plotted for the 5 CRE motifs showing the strongest positional bias in the 3 windows from -1 to -150 bp away from the TSS.

We performed a search of the literature to obtain information on the sequence of CREs in known CREB target genes. A total of 110 CREs in 93 known CREB target genes were retrieved. Although in some cases sequences longer than the 8 bp core motif were reported, only the core motifs were considered in this study. A total of 46 distinct CRE variants were identified. We searched a set of genomic sequences containing more than 46,000 human sequences spanning -1,000 bp to +1,000 bp with respect to the TSS, defined as "TSS spanning regions" (TSS-SRs) (see methods). The searches were performed on both the forward and reverse strands with respect to the direction of transcription. Using these sequences we computed representation index (RI) values in 40 × 50 bp windows along the TSS-SRs (see methods) for the 46 reported CRE variants and a non-CRE motif TGA**TA**TCA (TA-motif). The canonical CRE motif was highly over-represented in 3 adjacent 50 bp windows over the -1 to -150 bp region (mean RI_(-1 to -150) _8.7), but under-represented outside this region (mean RI_(outside -1 to -150) _0.8). The difference between these two mean RI values was used as a measure of positional bias towards the region from -1 to -150 bp. Positional bias values of all 47 motifs were computed and motifs with positional bias values greater than 2.6 standard deviations from the mean (P < 0.01) were classified as significant. Figure 1 shows the representation index (RI) profiles of 5 CRE motifs with the highest positional bias values and the non-CRE TA-motif. The canonical CRE and the TG-variant (TGA**TG**TCA) showed the highest positional bias (values 7.9 and 3.8, respectively) and were the only motifs to have significant positional bias. When considered separately, TG-variant motifs on the forward and reverse strand (with respect to the direction of transcription) were found to have similar positional bias values (data not shown). We selected the canonical CRE and the TG-variant to investigate the evolution of positional bias of CREs.

### Evolution of the positional bias of the canonical CRE

**Figure 2:**
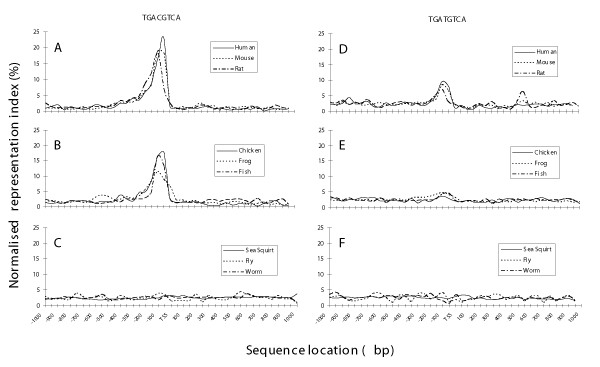
**Variation of representation index (RI) over the TSS-SR of 9 species**. The normalised representation index of each 50 bp window along the TSS-SRs are plotted for the canonical (TGACGTCA) and TG-variant (TGATGTCA) CREs in mouse, rat and dog (A and D); chicken, frog and fish (B and E); and sea squirt, fly and worm (C and F), respectively. In order to be comparable on the same scale, we normalised the RI values in each 50 bp by dividing them by the sum of all RI values in the 40 windows over the TSS-SRs. The values presented are percentages.

Since CREs have been described in a broad range of eukaryotes, we performed bi-directional searches for canonical and TG-variant CREs in the genomes of 9 organisms to see if the CRE positional bias is conserved. We computed RI values in 50 bp windows in the TSS-SR regions. Plots of normalized RI values of the canonical CRE versus distance from the TSS revealed a strong positional bias toward the TSS in the genomes of human (*H. Sapiens*), mouse (*M. musculus*), rat (*R. norvegicus*), chicken (*G. gallus*), frog (*X. tropicalis*) and zebrafish (*D. rerio*) (Figure 2A,B) but not in the genomes of the sea squirt (*C. intestinalis*), fruit fly (*D. melanogaster*) and worm (*C. elegans*) (Figure 2C). Positional bias was also observed for the TG-variant in human, mouse, and rat (Figure 2D). Little or no positional bias was observed for the TG-variant in chicken, frog and zebrafish (Figure 2E) and no positional bias was seen in the sea squirt, fruit fly and worm (Figure 2F). The CRE positional bias likely evolved after the separation of urochordata and vertebrata.

It would seem that selective conservation of canonical CREs located close to the TSS occurred during evolution. It is known that activated CREB is involved in the recruitment of other transcriptional co-activators, such as CREB binding protein, to promoter regions close to the TSS to facilitate transcription [30]. We postulate that this requirement for CREB to be in close proximity to the TSS might have imposed functional constraints on the location of the CRE and contributed to the evolution of CRE positional bias. We hypothesize that the positional bias of the TG-variant motif is a consequence of CpG to TpG transitions in canonical CREs due to cytosine methylation-deamination events. We explore this hypothesis in the following sections.

### Conservation of CREs in mammalian genomes

Using pair-wise comparisons, putative CREs from our survey in the human genome were checked for conservation of the motif sequence in mouse, rat and dog using the "LiftOver" tool [31,32]. Over 11,000 human CREs were mapped to orthologous sequences in mouse, rat or dog. The frequency of identical orthologous sequences found in mouse, rat or dog was used as a measure of conservation of the CRE motif variants. Table 1 shows the five most highly conserved CRE motifs between human and the other mammals. The canonical CRE had the highest motif conservation value (mean 57.0 ± 0.3%) and the TG-variant was the second most conserved (mean 33.5 ± 1.0%). Using the pair-wise comparisons of human canonical and TG-variant CREs with orthologous sequences in mouse, rat and dog, we investigated the frequency of occurrence of all possible octet motifs. Table 2 shows the five most frequently occurring motif pairs for human canonical and TG-variant CREs. We found that when human canonical CREs are not conserved in the other species they are most frequently the TG-variant (4.3 ± 0.3%) or its reverse complement, TGA**CA**TCA (CA-variant, 3.6 ± 0.8%) (Table 2). This is not surprising since, due to the high frequency of deamination of methylated cytosines in CpG dinucleotides to thymidine, CpG to TpG or CpA transitions are the most frequent DNA mutation in mammals [33]. Interestingly, the TA-motif, which can result from double-deamination of methylated CpG, was rare (0.2 ± 0.1%). Human TG-variants are most frequently found in the canonical form in the other mammals when they are not conserved (Table 2). Interestingly, for human TG-variant CREs, the third most frequent motif pair was the reverse complement, CA-variant CRE. Such differences may occur when the central CpG of an ancestral canonical CRE is methylated and deaminated on different strands during divergent evolution. In fact we identified 7 cases where canonical, TG-variant and CA-variant CREs are found in orthologous sequences in human, mouse, rat and dog. One of these was located 305 bp upstream of the TSS of human *CALCA*, a known CREB target gene. This CRE was canonical in human and rat, TG-variant in mouse and CA-variant in dog. The fourth most frequent motif pair in human TG-variant CREs was the non-functional TA-motif. These motif pairs could have been generated from an ancestral canonical CRE that was methyl-CpG deaminated on only one strand in human, but on both strands in the other species. Taken together these data show that both canonical and TG-variant CREs are highly conserved among mammals and that mutations involving CpG to TpG transitions are the most commonly occurring differences found in orthologous CREs.

### CRE methylation and CpG islands

Many mammalian promoters contain CpG islands, which are regions of high CpG density and hypomethylation. In order to investigate the frequency that CRE variants occur within CpG islands, we mapped the positions of human canonical and TG-variant CREs and the TA-motif onto the human CpG island track using the UCSC table browser data retrieval tool [34].

We found that 62% of human canonical CREs, but only 7% of TG-variant CREs and 2% of TA-motifs, were found in CpG islands. We looked at a subset of the canonical and TG-variant CREs that are conserved in at least one other mammal (mouse, rat or dog) and found that 81% of canonical, but only 13% of TG-variant CREs are located in CpG islands in human. Therefore, the CREs within the CpG islands appear to be more conserved. Since CpG islands are less methylated than other genomic DNA regions, CpG dinucleotides in CpG islands are less prone to spontaneous methyl-C deamination. This in turn may reduce the rate of mutation of canonical CREs in CpG islands to the TG- or CA-variants, or to the TA-motif. CREs that are outside of CpG islands are not protected from methylation and many of these may have been subject to deamination during evolution. We also found that the positional bias was stronger for both canonical and TG-variant CREs in CpG islands versus those not in CpG islands (data not shown).

To address the question of whether the rate of CpG to TpG transitions is different for CREs within or outside of the CpG islands, we analyzed the CpG island status of four subsets of human and mouse orthologous CREs: (1) CREs that were canonical in both human and mouse (CG:CG); (2) CREs that were TG-variants in both human and mouse (TG:TG); (3) CREs that were canonical in human, but TG-variants in mouse (CG:TG); and (4) CREs that were TG-variants in human, but canonical in mouse (TG:CG) (see Table 3). These sets were then partitioned according to whether the CRE pairs were or were not in a CpG island in human. We found that 80% of the CG:CG CRE pairs, but only 16% of TG:TG CRE pairs were in CpG islands in human. This is consistent with our findings above (see 'conservation of CREs in mammalian genomes'). Sixty percent of the CG:TG pairs and 32% of the TG:CG pairs were found in CpG islands. According to these results, when human canonical CREs are located outside a CpG island, they are twice as likely to be paired with TG-variants (CG:TG) than with canonical CREs (CG:CG) in mouse (40% and 20%, respectively). Similarly, the percentage of TG:CG pairs in human CpG islands is almost double the percentage of TG:TG pairs (32% and 17%, respectively). We also found that 80% of human canonical CREs that are conserved in mouse are in a CpG island in human versus only 57% of those not conserved in mouse. Taken together these data suggest that although CpG to TpG transitions within canonical CREs do occur in CpG island regions, the rates of transition are much higher when the CREs lie in non-CpG island regions.

Tissue specific methylation of canonical CREs has been shown to inhibit CREB binding [14] and methylation-dependant regulation of CREB activity has been demonstrated [35-38]. Most variants of the canonical CRE contain the motif TGACG, which are considered to be half-site CREs [1], and can also be methylated at the CpG. Such regulation, however, is not likely to function on TG-variant CREs since they lack the CpG dinucleotide that is the usual target of DNA methyltransferases in mammals. It has been established that TG-variant CREs are functional in a number of mammalian genes including *PLAT*, *Cga*, *RARA*, *RARB*, *NTS *and *CFTR *[25-29], but they may have lower specificity for CRE-binding proteins than canonical CREs [25]. Thus, a gene that is regulated by a canonical CRE in one species and a TG-variant in another species may show differences in basal, temporal or tissue specific gene expression. From the pair-wise comparisons of human CREs to orthologous sequences in other mammals we identified 99 human canonical CREs that were TG-variant CREs in at least one other species and 108 human TG-variant CREs that were canonical CREs in at least one other species (See Additional File 1). Further investigation is required to establish whether these putative CREs contribute to differences in gene expression that are relevant to human development or disease.

The appearance of heavily methylated genomes and CpG islands both coincide with the evolution of vertebrates [18,22]. CpG islands tend to associate with the 5'-end of genes, often spanning proximal promoter regions [22]. The urochordate, *Ciona intestalis*, has a fractionally methylated genome and shows examples of sequence resembling vertebrate CpG islands [39], which may hint at the beginning of the evolution of the CpG islands seen in vertebrates. We found that, unlike all the other chordates in our study, *Ciona *has no positional bias of the canonical CRE, suggesting that the positional bias of CREs evolved after the separation of vertebrates from the other chordates. Antequera [22] proposed a model for CpG island evolution in vertebrates that involves protection of CpG island regions from DNA methylation by the initial assembly of the replication machinery. Since vertebrate CpG islands are hypomethylated regions in a sea of CpG methylation, it follows that the evolution of this feature was dependent on the evolution of genome-wide methylation. Most of the canonical CREs identified in this study were found to be located within CpG islands. We propose that, in addition to the selective pressure for CREs to be in close proximity to the TSS, the evolution of positional bias of canonical CREs was also assisted by the maintenance of CpG islands in a hypomethylated state in vertebrates. CREs that lie outside of the boundaries of CpG islands are more likely to be methylated and are vulnerable to methyl-cytosine deamination to thymidine. Our finding that most TG-variant CREs, which can be formed by CpG to TpG transition in canonical CREs, were found outside CpG islands supports this hypothesis.

We speculate that early vertebrates had many more canonical CRE motifs distributed throughout the genome than today's vertebrates and that genome-wide methylation and deamination of methyl-CpG has removed many of these motifs from vertebrate genomes. A whole genome survey showed that the human genome contains only 25% of the expected number of canonical CRE motifs (data not shown). CpG islands may have preserved CREs (especially canonical CREs) as functional transcription factor binding sites in regions close to the core promoter. The result of this process is the observed positional bias of the canonical CRE. CpG islands however are not necessarily stable over time. There is evidence to suggest that mouse CpG islands have eroded [40]. Evolutionary changes in the CpG island status of genes could leave canonical CREs exposed to methylation and trigger conversion to the TG-variant. Also, CpG islands are not devoid of methylation. Canonical CREs in CpG islands can still be methylated, in some cases in a tissue dependent or temporal manner. In fact, we found that 12 out of 26 canonical CREs recently identified as always methylated or differentially methylated [14] were located in CpG islands. We further speculate that the weaker positional bias observed in the TG-variant is simply a consequence of deamination of methyl-C in the core of the canonical CREs.

## Conclusion

In summary, our data suggest that a positional bias of canonical CREs towards the TSS evolved in vertebrates after the separation from urochordates. The weaker positional bias observed in the TG-variant is likely a consequence of deamination of methyl-C in the core of the canonical CREs. The canonical CRE is the most highly conserved CRE variant in mammals and shows the strongest positional bias toward the TSS. The most frequent mutations in canonical CREs are likely due to methyl-C deamination events. We observed that most canonical CREs lay within CpG islands where they are likely maintained in an un-methylated state, thus protecting them from methyl-C deamination. Conversely, TG-variant CREs were generally found in non-CpG island regions. Many of these TG-variant CREs may have been formed from canonical CREs through methyl-C deamination. Our discovery of orthologous CREs with different dynamic methylation potential amongst mammals may help to uncover important differences in the regulation of orthologous CREB target genes relevant to human development and disease. In future work we would like to extend our study to other transcription factors that show positional bias and bind to sites with methylation-sensitive CpG dinucleotides.

## Methods

### Sources of the sequences

The sequence spanning -1,000 bp to +1,000 bp with respect to the TSS, defined as "TSS spanning region" (TSS-SR), was chosen for analysis in this study. For the study of CpG → TpG transitions in orthologous genes a comprehensive search for CREs was first performed in human on both strands of the TSS-SRs. Since genes are frequently represented by multiple sequences with different TSSs, a database of human TSS-SRs was generated comprising all sequences with a defined TSS from H-inv [41], RefSeq [42] and the Mammalian Gene Collection (MGC, [43]). The sequences were obtained from the UCSC genome database by means of the table browser data retrieval tool [34] using UCSC genome version hg17. Only sequences with unique TSS positions that are different from the genomic positions of the start codon of corresponding genes were considered. In this dataset, many genes are represented by multiple TSS-SRs due to the presence of alternate TSSs in the source databases. The numbers of sequences obtained from each source and those in the final dataset are summarized in Table 4.

Sequences from 9 species, *Homo sapiens*, *Mus musculus*, *Rattus norvegicus*, *Gallus gallus*, *Xenopus tropicalis*, *Danio rerio*, *Ciona intestinalis*, *Drosophila melanogaster *and *Caenorhabditis elegans*, were obtained from the UCSC genome database in a similar manner. However, for each species only a single source was used and the sequences were not filtered for redundancies. Table 5 summarizes the source and the number of TSS-SRs for each species.

### Measuring motif positional bias

The representation index (**RI**) was used to measure representation of a motif in a set of sequences and was defined as the total number of occurrences of a motif (**N**) divided by the statistical expectation value (**E**) of the motif in a given set of sequences [44]. Motif expectation values were calculated using the following formula:

***E ***= ***p*(Mf) **× (Sequence Length - *n*+1) × (number of sequences)

Where ***p*(Mf) **= ∏***p*(*x*_*i*_) **{*i *= *1, 2, ......, n*}, **Mf **is a motif; ***p*(Mf) **is the motif probability; ***p*(*x*_*i*_) **is the probability of each base in a motif calculated using the average contents of A, C, G, T respectively in each 50 bp window of the sequence set; *x*_*i *_is the base at position *i*; and *n *is the length of the motif. In this study, RI values were calculated for each of the 40 × 50 bp windows along the TSS-SR (-1,000 to +1,000 bp). Motif searches and RI value calculations were performed using BioMiner, a suite of data mining tools designed and built in house.

Positional bias in the region from -1 to -150 bp with respect to the TSS was defined as the difference in mean RI values between the 3 × 50 bp windows from -1 to -150 bp and the other 37 × 50 bp windows. The positional bias of a motif was classified as significant if the value was greater than 2.6 standard deviations (p < 0.01) from the mean of the positional bias values of all motifs.

### Verification of CREs by cross-species sequence comparison

LiftOver [31], a tool at UCSC for conversion of genome coordinates between assemblies either within a species or between species, was used to convert the genomic coordinates of all human CREs to mouse, rat and dog coordinates. Sequences at these coordinates were then interrogated for the presence of a CRE in each target species. The cross-referenced CREs are referred to as orthologous CREs in this study.

## Authors' contributions

BS, HF and YP designed the study, developed the methods, analysed and interpreted the data, and drafted the manuscript. PRW and MS participated in the design of the study and revised the manuscript. FAF participated in the development of the software that was used and revised the manuscript.

## Supplementary Material

Additional file 1 **CREs that are canonical in human and TG-variant in mouse rat or dog and vice-versa**. This excel file contains details on the CREs that were canonical in human and TG-variant in mouse, rat, or dog and vice-versa. The table includes: the genomic location and strand (+/-) of the human TSS-SR in which the CRE was found; the position (bp) of the CRE relative to the TSS; the accession number, Entrez Gene id, symbol and name of the gene associated with the TSS-SR; the genomic location of the CRE in human, and the orthologs in mouse, rat and dog; the sequence (with respect to the TSS-SR strand) of the human CRE and the orthologous motifs in mouse, rat and dog; a summary of the CRE type in each species (Canonical, TG-variant, CA-variant, Half-site, TA-motif or none); the CpG island status of the human CRE (Yes : in a CpG island, No : not in a CpG island). Click here for file

## Figures and Tables

**Table 1:** Conservation of human CREs in mouse, rat, and dog

**CRE Motif**	**Percentage of sequences conserved**			**Mean**	**SD**
			
	**Mouse**	**Rat**	**Dog**		
TGACGTCA	56.9	57.2	56.7	57.0	0.3
TGATGTCA	34.6	32.9	33.0	33.5	1.0
TGACGCAC	26.3	27.0	33.0	28.8	3.7
TGACGTCG	27.2	27.9	31.2	28.8	2.2
TGACGTGG	29.4	29.9	24.7	28.0	2.9

**Table 2:** Frequency of orthologous sequences

**In human**	**In mouse, rat or dog**	**Number of occurrences**				**Mean percentage of all occurrences**	**SD**
				
		**Mouse**	**Rat**	**Dog**	**Mean**		
	TGACGTCA	373	364	380	372.3	56.9	0.3
	TGA**T**GTCA	26	27	31	28.0	4.3	0.3
TGACGTCA	TGAC**A**TCA	29	22	19	23.3	3.6	0.8
	TGACG**C**CA	13	14	8	11.7	1.8	0.5
	TGACGTC**G**	7	10	12	9.7	1.5	0.4

	TGATGTCA	475	438	540	484.3	33.5	1.0
	TGA**C**GTCA	44	47	57	49.3	3.4	0.2
TGATGTCA	TGA**CA**TCA	24	23	25	24.0	1.7	0.1
	TGAT**A**TCA	17	20	29	22.0	1.5	0.3
	T**T**A**C**GTCA	18	20	20	19.3	1.3	0.1

Sites differing from the human sequence are highlighted in bold.

**Table 3:** Canonical and TG-variant CREs in human CpG islands

**In human**	**In mouse**	**human:mouse pair**	**Number of occurrences**		**Percentage in CpG islands of all occurrences**
				
			**In CpG island**	**Total**	
TGA**CG**TCA	TGA**CG**TCA	CG:CG	298	373	80
	TGA**TG**TCA	CG:TG	33	55	60

TGA**TG**TCA	TGA**CG**TCA	TG:CG	14	44	32
	TGA**TG**TCA	TG:TG	82	475	17

**Table 4:** Summary of human (hg17) TSS-SRs

**RefSeq**	**MGC**	**hINV**	**Sequences with unique genomic position w.r.t. the TSS**
21,164	18,016	23,903	46,485

RefSeq: Sequences from NCBIs RefSeq collection, MGC: Sequences from the Mammalian Genome Collection, hINV: Sequences from the human invitational clone database

**Table 5:** Summary of TSS-SRs from 9 species

**Species**	**UCSC Genome version**	**UCSC Data Table**	**Number of sequences**
*Homo sapiens*	hg17	RefSeq Genes	24,819
*Mus musculus*	mm5	RefSeq Genes	20,199
*Rattus norvegicus*	rn3	RefSeq Genes	8,555
*Gallus gallus*	galGal2	RefSeq Genes	1,692
*Xenopus tropicalis*	xenTro1	JGI genes	33,749
*Danio rerio*	danRer2	RefSeq Genes	9,944
*Ciona intestinalis*	ci1	JGI genes	15,569
*Drosophila melanogaster*	dm1	RefSeq Genes	18,960
*Caenorhabditis elegans*	ce2	RefSeq Genes	23,461
